# Detection of Increased Relative Expression Units of *Bacteroides* and *Prevotella*, and Decreased *Clostridium leptum* in Stool Samples from Brazilian Rheumatoid Arthritis Patients: A Pilot Study

**DOI:** 10.3390/microorganisms7100413

**Published:** 2019-10-01

**Authors:** Guilherme S. P. Rodrigues, Leonardo C. F. Cayres, Fernanda P. Gonçalves, Nauyta N. C. Takaoka, André H. Lengert, Aline Tansini, João L. Brisotti, Carolina B. G. Sasdelli, Gislane L. V. de Oliveira

**Affiliations:** 1 Microbiome Study Group, School of Health Sciences Dr. Paulo Prata, Barretos, São Paulo 14785-002, Brazil; guisprodrigues@gmail.com (G.S.P.R.); leonardo_cayres_2608@hotmail.com (L.C.F.C.); fernanda_fpg@hotmail.com (F.P.G.); nauytatakaoka@gmail.com (N.N.C.T.); 2 Molecular Oncology Research Center, Barretos Cancer Hospital, Barretos, São Paulo 14784-400, Brazil; ahlengert@gmail.com (A.H.L.); aline.cpom@hcancerbarretos.com.br (A.T.); 3 Barretos Medical Specialties Outpatient (AME), Barretos, São Paulo 14785-000, Brazil; jlbrisotti@gmail.com (J.L.B.); carollgarciasp@yahoo.com.br (C.B.G.S.); 4 Microbiology Program, Institute of Biosciences, Humanities and Exact Sciences (IBILCE), São Paulo State University (UNESP), Sao Jose do Rio Preto, São Paulo 15054-000, Brazil

**Keywords:** rheumatoid arthritis, disease modifying antirheumatic drugs, diet, gut bacteria, cytokines

## Abstract

Interactions between gut microbes and disease modifying antirheumatic drugs (DMARDs) have been proposed. The aim of the present study was to evaluate the presence of some specific bacteria in stool samples from Brazilian RA patients receiving DMARDs and correlate these data with diet, clinical parameters, and cytokines. Stool samples were used for gut bacteria evalutation by qPCR. Serum samples were used to quantify IL-4 and IL-10 by flow cytometer. Statistics were performed by Pearson chi-square, Mann–Whitney U test, and Spearman’s correlation. The study included 20 RA patients and 30 healthy controls. There were no significant differences (*p* > 0.05) in dietary habits between RA patients and controls. Concerning gut bacteria, we observed an increase in relative expression units (REU) of *Bacteroides* and *Prevotella* species in stool samples from patients, and a decrease in REU of *Clostridium leptum* when compared with healthy controls. Positive correlation between *Prevotella* and rheumatoid factor was detected. The IL-4 and IL-10 concentrations were increased in patients when compared with controls. We concluded that gut bacteria are different between RA patients receiving DMARDs and healthy controls. Further studies are necessary to determine the real role of gut microbes and their metabolities in clinical response to different DMARDs in RA patients.

## 1. Introduction

Rheumatoid arthritis (RA) is a systemic autoimmune disease, mediated by immune reactions against synovial proteins, promoting chronic inflammation, and bone and cartilage damage [1]. The disease predominantly affects women between 20 and 50 years, and is associated with disability, sick leave, loss of productivity, and poor quality of life [2,3]. The worldwide RA prevalence reaches about 5 people per 1000 adults, and was estimated as affecting between 0.2% and 1% of the Brazilian population [2,4]. The disease incurs a significant financial burden to patients, society, and national economies. In the United States, the total health costs are estimated at $41.6 billion per year, and in Europe, the direct/indirect healthcare to treat RA patients is approximately €45 billion per year [3,5]. The Brazilian Unified National Health System (SUS) spends approximately BRL 113,900.00/patients during the 48 months of methotrexate (MTX) monotherapy, and about BRL 10 million/patients (≈2.5 million dollars) with refractory patients that used MTX and infliximab since the beginning of the treatment [6].

RA development involves genetic and environmental factors, and the increased mortality is associated with systemic complications, such as involvement of the lungs, kidneys, and heart [7]. Cardiovascular diseases in RA patients are the major causes of mortality, around 1.5 times higher than in the general population [8]. The RA etiopathogenesis are complex and involve rheumatoid factor and anticitrullinated antibodies, which are detected in blood before RA diagnosis, suggesting that autoimmunity might be generated at distant sites from the joints, including the oral–gastrointestinal mucosa [7,9]. Furthermore, the low concordance rate in twin studies points to the importance of environmental factors, including smoking, infections, diet, and oral/intestinal dysbiosis [10].

Studies in animal models suggest that the gut microbiota affects innate and adaptive immunity, and plays roles in local and systemic inflammation, triggering joint damage [11]. Experiments in collagen-induced arthritis (CIA) mice showed prevalence of *Desulfovibrio*, *Prevotella*, *Parabacteroides*, *Odoribacter*, *Acetatifactor*, *Blautia*, *Coprococcus*, and *Ruminococcus* genera, and increased IL-6, IFN-γ, and IL-17 cytokines when antibiotics were administered [12]. Additionally, previous studies showed prevalence of *Clostridia* species in fecal samples, as well as increased intestinal permeability and Th17 profile in arthritis-susceptible mice [13]. Furthermore, the fecal transplantation from RA patients to germ-free arthritis-prone SKG mice induces the Th17 profile in the gut mucosa and severe RA, and when SKG dendritic cells were cultivated with *Prevotella copri*, there was an increased IL-17 response to RA autoantigens, suggesting that the gut microbes could induce autoreactive cells in the gut mucosa [14]. Interestingly, although MTX induces a decrease in bloodstream inflammation, MTX-treated CIA mice showed a decrease in microbial diversity, expansion of *Prevotella* spp., and no association with eubiotic microbiome [15,16].

In humans, researchers reported the prevalence of *Prevotella* species in newly diagnosed arthritic patients, and increased *Eggerthella*, *Actinomyces*, *Turibacter*, *Streptococcus,* and *Collinsela* genera with positive association with IL-17 cytokine [17,18]. Moreover, decreased alpha-diversity of the gut microbiota was detected in RA patients when compared with the control group. The C-reactive protein, rheumatoid factor levels, disease progression, and MTX therapy positively correlated with beta-diversity in RA patients, suggesting that the treatment may affect the interactions between microbiota and mucosal immune cells in the gut, and supporting the hypothesis that gut microbes and their metabolities may interfere in the clinical response to disease-modifying antirheumatic drugs (DMARDs) [19,20].

On the basis of this background and the fact that there are no studies evaluating the gut bacteria in Brazilian RA patients, the aim of the present study was to evaluate the presence of some specific bacteria in stool samples from Brazilian RA patients receiving DMARDs, and correlate these data with diet, clinical parameters, and cytokines.

## 2. Materials and Methods

### 2.1. Study Population

RA patients, diagnosed according to the American College of Rheumatology (ACR)/European League Against Rheumatism (EULAR) criteria [21], were enrolled by the physician from the Rheumatology Department from Barretos Medical Specialties Outpatient (AME-Barretos), Sao Paulo, Brazil. The present study was approved by the Barretos Cancer Hospital Ethics Committee (Process number 1269/2016), and informed consent was obtained from RA patients and control subjects. A total of 20 RA patients ranging from 36 to 71 years of age (mean age ± standard deviation (SD) = 56.2 ± 9.4 years) were included. The disease activity score (DAS) was calculated by DAS28-CRP3, which includes swollen and tender joint count and C-reactive protein (CRP) levels. Table 1 summarizes demographic and clinical parameters of the RA patients. A total of 30 healthy controls (93.3% females; 80% Caucasian, 16.6% Afro-descendant, 3.33% Hispanic), without RA family history, ranging from 25 to 70 years of age (mean age ± SD = 51.8 ± 12.9 years), were enrolled for the study.

Exclusion criteria for both groups included the use of antibiotics and laxatives in the last 20 days, vaccination in the last 30 days, gastrointestinal surgeries, inflammatory bowel diseases, and chronic/acute diarrhea. Controls that used anti-inflammatories in the last 20 days or immunosuppressive drugs in the last 30 days were also excluded from this study.

At enrollment, RA patients and control subjects answered a survey regarding dietary habits, such as consumption of vegetables, fruits, carbohydrates, animal-derived proteins, trans fats, milk and derivatives, hot drinks (coffee and tea), canned food, condiments, and spicy food. The consumption frequency was expressed as never consumes, rarely consumes (less than once a month/1–3 times a month/1–2 times a week), and frequently consumes (most days, but not every day/every day). Thereafter, 10 mL of peripheral blood was collected in Gel BD SST II Advance tubes (BD Biosciences, CA, USA), and serum samples were stored at −80 °C until cytokine quantification. Stool samples were delivered by patients/controls within 3 to 5 days after blood collection and were stored at −20 °C until DNA extraction. DNA extraction was performed within 5 days after stool sample delivery.

### 2.2. DNA Extraction and Real-Time PCR

Bacterial DNA was extracted from 200 mg of stool samples by using QIAamp DNA Stool Mini Kit (QIAGEN, CA, USA), according to the manufacturer instructions. The presence of specific groups of bacteria was determined by using primers described previously, and the genus-specific primers were designed using 16S rRNA gene sequences from the Ribosomal Database Project (RDP 10) [22]. Primers were specific for *Bacteroides* (*Bac*), *Bifidobacterium* (*Bif*), *Clostridium coccoides* (*Ccoc*), *Clostridium coccoides-Eubacteria rectale* (*CIEub*), *Clostridium leptum* (*Clept*), *Lactobacillus* (*Lac*), *Prevotella* (*Prev*), and *Roseburia* (*Ros*). Reactions were performed by using Power SYBR Green PCR Master Mix (Applied Biosystems, Life Technologies, CA, USA), 2 µM of forward and reverse primers, and 5 ng of DNA. Negative controls without DNA samples were included in each experiment. For relative quantification, DNA copy numbers from target primers were normalized for the copy numbers of universal primer (*Univ*). The relative expression units (REU) were calculated by using cycle threshold (Ct) values [23], and in the present work, was expressed as REU per 200 mg of stool. These data were graphically represented in Log, base 2 (Log 2).

### 2.3. Cytokine Quantification by Flow Cytometer

Peripheral blood was collected in Gel BD SST II Advance tubes (BD Biosciences, CA, USA), and serum samples were isolated by centrifugation at 1.372× *g* for 5 min at 25 °C. IL-4 and IL-10 concentrations were detected by flow cytometer FACSCanto II (BD Biosciences, CA, USA), using the cytometric bead array kit (BD Biosciences, CA, USA). The analyses were performed by using BDFCAP array software and data were presented as pg/mL.

### 2.4. Statistical Analysis

Data from the dietary surveys were analyzed by Pearson’s chi-square test by using IBM SPSS Statistics, version 20, and the results underwent a Benjamini–Hochberg post-test correction by using InVivoStat version 3.7. The comparisons between the relative expression units of the specific bacterial groups and the serum concentrations of IL-4 and IL-10 were analyzed by nonparametric Mann–Whitney U test. Correlations between the relative expression units of the gut bacteria, dietary habits, and cytokine concentrations were performed by Spearman’s correlation. Normality test, Mann–Whitney U test, and Spearman’s correlation were calculated by using GraphPad software, Prism version 8.0.1. *p* < 0.05 was considered statistically significant.

## 3. Results

### 3.1. Increased Relative Expression Units of Bacteroides and Prevotella, and Decreased Clostridium leptum in the Gut Bacteria of RA Patients

**Figure 1:**
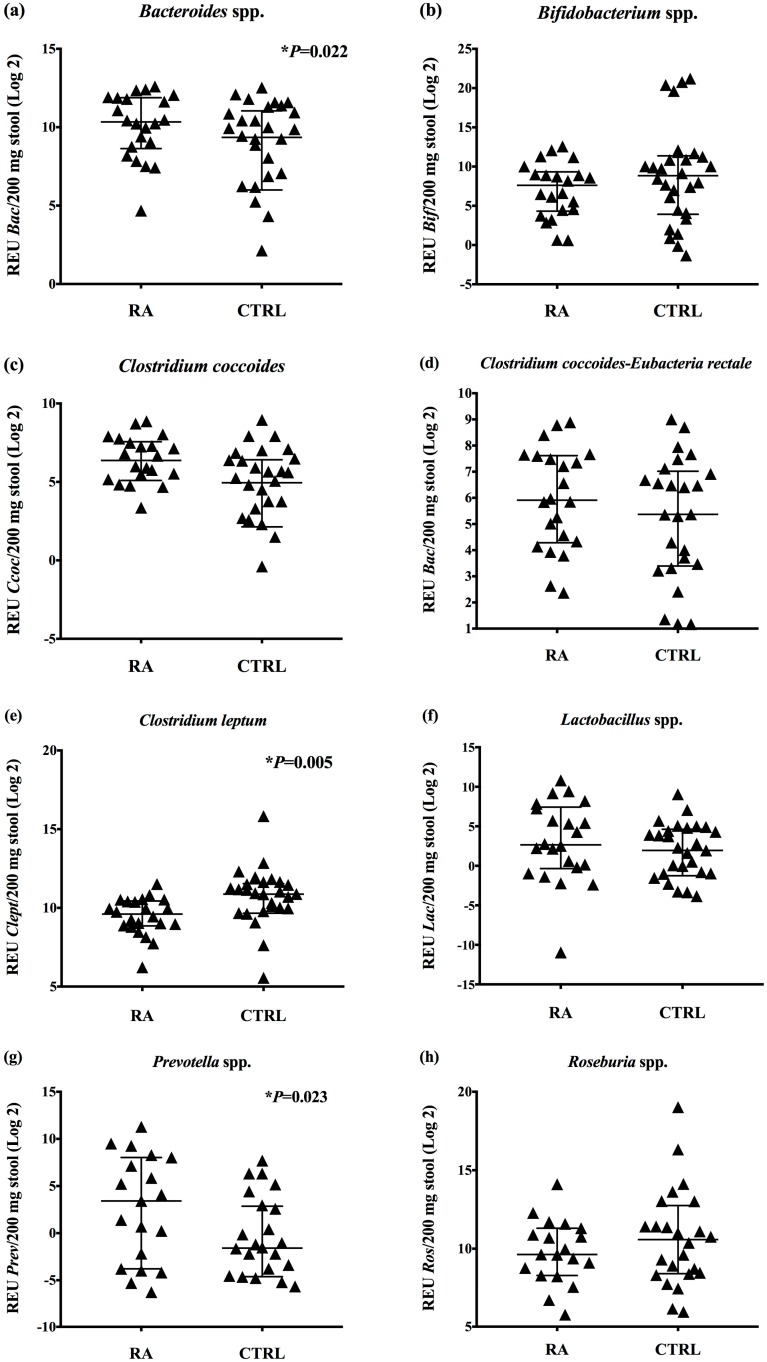
Relative expression units of gut bacteria found in stool samples from patients (RA) receiving DMARDs, and healthy controls (CTRL). (**a**) *Bacteroides* species, (**b**) *Bifidobacterium* species, (**c**) *Clostridium coccoides*, (**d**) *Clostridium coccoides-Eubacterium-rectale*, (**e**) *Clostridium leptum*, (**f**) *Lactobacillus* species, (**g**) *Prevotella* species, and (**h**) *Roseburia* species. Bars represent the median with interquartile range of relative expression units (REU) per 200 mg of stool, and they were graphically represented in Log, base 2 (Log 2). Mann–Whitney U test analysis was used. * *p* < 0.05.

To evaluate the gut bacteria in RA patients receiving DMARDs, we analyzed the presence of some specific bacterial groups in stool samples by real-time PCR. We observed a significant increase in the relative expression units of *Bacteroides* and *Prevotella* species in stool samples from RA patients (median *Bac*: 1294; *p* = 0.022; median *Prev*: 10.66; *p* = 0.023) when compared with healthy controls (median *Bac*: 654.9; median *Prev*: 0.335) (Figure 1a,g). On the other hand, we detected a significant decrease in relative expression units of *Clostridium leptum* in RA patients (median: 779.8; *p* = 0.005), compared with control subjects (median: 1872) (Figure 1e). Beyond that, there were no statistically significant differences (*p* > 0.05) in relative expression units of *Bifidobacterium* (median: 195.7), *Clostridium coccoides* (median: 82.78), *Clostridium coccoides-Eubacteria rectale* (median: 60.17), *Lactobacillus* (median: 6.31), and *Roseburia* species (median: 795.1) in stool samples from RA patients, compared with controls (median *Bif*: 457.5; *Ccoc*: 48.86; CIEub: 41.37; *Lac*: 3,888; *Ros:* 1.535) (Figure 1).

Moreover, when we classified the patients in moderate–severe RA (DAS28-CRP3  > 3.2; *N* = 16) and mild disease (DAS28-CRP3 < 3.2; *N* = 3), there were no significant differences (*p* > 0.05) in relative expression units of *Bacteroides*, *Bifidobacterium*, *Clostridium coccoides, Clostridium coccoides-Eubacterium rectale, Clostridium leptum, Lactobacillus, Prevotella*, and *Roseburia* in stool samples from RA patients. Likewise, when we classified RA patients by non-steroidal anti-inflammatories (NSAIDs)/DMARDs (*N* = 13) versus biologic DMARDs (adalimumab/abatacept) therapies (*N* = 6), there were no significant differences (*p* > 0.05) in the relative expression units of *Bacteroides*, *Bifidobacterium*, *Clostridium coccoides, Clostridium coccoides-Eubacterium rectale, Clostridium leptum, Lactobacillus, Prevotella*, and *Roseburia* between the evaluated groups.

### 3.2. Dietary Habits and Correlations with the Gut Bacteria in RA Patients

To access the dietary habits of the RA patients and controls, we applied a survey concerning the frequency of consumption of vegetables, fruits, carbohydrates, animal-derived proteins, trans fats, milk and derivatives, hot drinks, canned food, condiments and spicy food (Table 2). The interviewees reported the regular consumption of vegetables (patients (RA) = 75%; controls (C) = 80%), fresh fruits P = 75%; C = 60%), carbohydrates (RA = 70%; C = 70%), animal-derived proteins (RA = 60%; C = 60%), trans fats (RA = 25%; C = 20%), dairy products (RA = 65%; C = 66.7%), hot drinks (RA = 95%; C = 76.7%), canned products (RA = 10%; C = 10%), condiments (RA = 5%; C = 0%), and spicy food (RA = 50%; C = 10%). When we compared the diet between RA patients and controls, there were no significant differences (*p* < 0.05) in any of the evaluated variables.

To find correlations between dietary habits and gut bacteria found in RA patients, we used the consumption frequencies and the relative expression units of bacterial groups detected in stool samples. We observed a positive correlation (*p* = 0.04; *r* = 0.26) between animal-derived protein consumption and the relative expression units of *Prevotella* species. Furthermore, we found a negative correlation between dairy products intake and the relative expression units of *Bacteroides* species (*p* = 0.04; *r* = −0.27). Furthermore, we detected a positive correlation between trans fat intake and the relative expression units of *Bifidobacterium* (*p* = 0.02; *r* = 0.30) and *Roseburia* (*p* = 0.04; *r* = 0.26). The consumption of hot drinks negatively correlated with relative expression units of *Bifidobacterium* (*p* = 0.03; *r* = −0.28), *Roseburia* (*p* = 0.03; *r* = −0.29), and *Clostridium leptum* (*p* = 0.03; *r* = −0.28).

### 3.3. Correlations between the Gut Bacteria and Clinical Data

**Figure 2:**
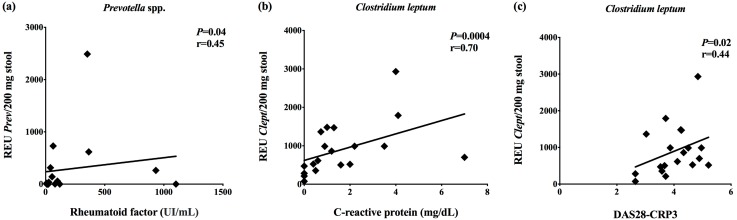
Spearman’s correlation between the relative expression units (REU) of the gut bacteria and clinical data. (**a**) Relative expression units of *Prevotella* species and rheumatoid factor concentrations, (**b**) REU of *Clostridium leptum* and C-reactive protein levels, and (**c**) REU of *Clostridium leptum* and the disease score DAS28-CRP3.

We found a positive correlation between the relative expression units of *Prevotella* species in stool samples from RA patients and serum concentrations of rheumatoid factor (*p* = 0.04; *r* = 0.45) (Figure 2a). The relative expression units of *Clostridium leptum* positively correlated with C-reactive protein levels (*p* = 0.0004; *r* = 0.70) and DAS28-CRP-3 score (*p* = 0.02; *r* = 0.44) (Figure 2b,c). There were no correlations among relative expression units of *Bacteroides*, *Bifidobacterium*, *Clostridium coccoides*, *Clostridium coccoides-Eubacterium rectale*, *Clostridium leptum*, *Lactobacillus*, *Prevotella*, and *Roseburia* species with erythrocyte sedimentation rate and disease duration.

### 3.4. Increased Serum Concentrations of IL-4 and IL-10 in RA Patients

**Figure 3:**
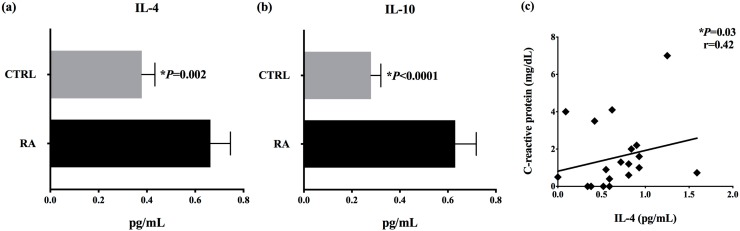
Cytokine concentrations (pg/mL) in patients (RA) and healthy controls (CTRL), and correlation with clinical data (Mann–Whitney U test). (**a**) IL-4 serum concentration, (**b**) IL-10 serum concentration, (**c**) positive Spearman’s correlation between IL-4 serum concentration and C-reactive protein levels (mg/dL).

In order to determine the serum concentrations of anti-inflammatory cytokines in RA patients receiving DMARDs, we quantified IL-4 and IL-10 by cytometric bead array. There were significant differences (*p* < 0.05) in concentrations of IL-4 and IL-10 in patients’ serum (mean ± standard error IL-4: 0.3239 ± 0.0743 pg/mL; IL-10: 0.265 ± 0.0429 pg/mL) when compared with controls (IL-4: 0.2839 ± 0.2244 pg/mL; IL-10: 0.2422 ± 0.18 pg/mL) (Figure 3a,b). We found a positive correlation between IL-4 serum concentrations and C-reactive protein levels in RA patients (*p* = 0.03; *r* = 0.42) (Figure 3c). There were no correlations between IL-4 and IL-10 serum concentrations and the relative expression units of *Bacteroides*, *Bifidobacterium*, *Clostridium coccoides*, *Clostridium coccoides-Eubacterium rectale*, *Clostridium leptum*, *Lactobacillus*, *Prevotella*, and *Roseburia* detected in stool samples from RA patients.

## 4. Discussion

According to recent studies, there is a possibility that autoimmune reactions start at mucosal surfaces and are influenced by gut microbes [9]. Some evidence related to RA etiopathogenesis include: (a) Some gut microbes have an arthritogenic effect when fragments are intravenously administered in mice, different to that occurring in germ-free conditions [17,24]; (b) intestinal dysbiosis has been detected in RA patients in several studies [24,25,26,27,28,29], including in early diagnosed RA, with increased Gram-negative *Prevotella* species and decreased *Bifidobacterium* species [14,17,18]; (c) Dysbiosis in mucosal sites may induce tolerance breakdown to citrullinated antigens, and the autoantibodies found in RA patients recognize citrullinated epitopes in antigens derived from the gut microbes [30,31]; (d) dietary habits can shape the gut microbiota composition and may influence the inflammatory markers in RA patients [32,33,34]; (e) some disease-modifying drugs present antimicrobial activity, and can restore the gut microbiome in patients with clinical response to these DMARDs [19,20]. On the basis of this evidenc, our aim relies on evaluating the presence of some specific bacteria in stool samples from Brazilian RA patients, receiving DMARDs, and correlating these data with diet, clinical parameters, and cytokines.

As discussed earlier, diet can shape the gut microbiota and influence the inflammatory markers in RA patients [32,33,34]. One of these previous studies concluded that vegetarianism can affect the gut microbiota composition in RA patients and could be associated with improvements in disease activity [34]. In our study, there are no significant differences in dietary habits between patients and controls, but we detected correlations between animal-derived protein consumption and *Prevotella* species, dairy products and *Bacteroides* species, trans fat intake and *Bifidobacterium*, and *Roseburia* species in RA patients, but no correlations between diet and inflammatory markers in RA patients. Wu et al. (2011) evaluated dietary habits and gut microbiota in 98 healthy volunteers, and showed that *Bacteroides* spp. were associated with the consumption of animal proteins and saturated fat, while was *Prevotella* correlated with carbohydrates and simple sugar intake [35].

Concerning gut bacteria, we detected an increase in relative expression units of *Bacteroides* and *Prevotella* species in stool samples from Brazilian RA patients (*N* = 20), and a decrease in *Clostridium leptum*, when compared with healthy controls (*N* = 30). By using the same technology as our work (qPCR), Liu et al. (2013) evaluated 15 patients with early RA and demonstrated that fecal microbiota of these patients presented increased absolute copy numbers of *Lactobacillus salivarius*, *Lactobacillus iners*, and *Lactobacillus ruminis* compared with the healthy controls (*N* = 15) [29]. By using 16S technologies, Maeda and Takeda (2017) showed that about one-third of newly-diagnosed RA patients (*N* = 17) presented higher abundance of *Prevotella copri* in the gut, when compared with controls (*N* = 14) [14]. Also, Scher et al. (2013) evaluated newly diagnosed RA patients (NORA group = 44) or chronic RA patients using DMARDs (CRA group = 26). The study showed increased abundance of *Prevotella copri* in the NORA group, and a significant increase in *Bacteroides* and a decrease in *Prevotella* species in the CRA group, when compared with the control group (*N* = 28) [17]. By using this previous Scher work and module networks to identify cause-and-effect relationships, Lu et al. (2017) demonstrated that the NORA dysbiotic group is connected to later MTX treated-patients, and NORA eubiotic to prednisone ones, suggesting that the previous eubiotic or dysbiotic condition is predictive of the severity of the disease and of the associated therapy [36].

Researchers have also identified a gut microbiota signature in RA patients, with decreased alpha-diversity, that positively correlated with increased rheumatoid factor and disease progression [18]. Prediction models showed that *Collinsella*, *Eggerthella*, and *Faecalibacterium* segregated with RA, along with *Collinsella* abundance, positively correlated with IL-17 inflammatory cytokine [18]. The *Eggerthella* and *Collinsella* abuncances were not associated with MTX, prednisone, and hydroxychloroquine [18]. In this work, MTX or hydroxychloroquine treated-patients presented an increase in species richness and diversity, suggesting the possible recovery of healthy gut microbiota after treatment [18]. The role of MTX in the gut microbiota is still a controversial field, and data from animal models showed that rats treated with MTX developed mucositis and presented decreased global microbial abundance, especially in anaerobes, diarrhoea, and damaged villous in the small intestine [37,38,39]. Another study, performed by Zhou et al. (2018), showed that the gavage of MTX-treated mice with an anti-inflammatory *Bacteroides fragilis* improved the inflammatory condition and decreased macrophage M1 polarization, supporting the idea that gut microbiota have an important impact on MTX-induced intestinal mucositis [40].

On the basis of evidence that there are reciprocal interactions between drugs and gut microbiota, Picchianti-Diamanti et al. (2018) evaluated the effect of DMARDs in gut microbiota from RA patients [19]. First of all, authors detected dysbiosis in RA patients and a significant decrease in *Faecalibacterium* genus and *Faecalibacterium prausnitzii* in the gut microbiota from naïve RA patients (*N* = 11) when compared with healthy controls (*N* = 10) [19]. They also detected a decrease in relative abundance of Enterobacteriales in MTX-treated patients (*N* = 11), decrease in Deltaproteobacteria and Clostridiaceae in the etanercept-treated group (ETN, *N* = 10), and no significant differences in ETN with MTX therapy (*N* = 10) when compared with naïve RA patients [19]. Authors concluded that the anti-TNF therapy is able to modulate the gut microbiota and partially restore the beneficial microbes [19]. Another study, using metagenomic shotgun sequencing and metagenome-wide association study of fecal, dental, and salivary samples from naïve RA patients (*N* = 77), DMARD-treated patients (*N* = 21) and healthy controls (*N* = 80), showed that the oral and gut dysbiosis associated with RA could be partially restored by DMARD treatment [20]. Specifically, MTX was shown to modify oral/gut microbiota composition and partly reestablish a healthy RA microbiome [20]. In this descriptive pilot study, we found significant differences in gut bacteria from RA patients receiving DMARDs when compared with healthy controls. Although our study presents limitations regarding the number of enrolled patients and the methodology used to study microbial groups, there are no studies in existence that evaluate the gut bacteria in Brazilian RA patients. Furthermore, we showed a positive correlation between the increased relative expression units of *Prevotella* species and rheumatoid factor levels in RA patients, suggesting the possible role of gut microbes and their metabolities in response to DMARDs [19,20,36].

Moreover, we detected decreased relative expression units of *Clostridium leptum* in RA patients when compared with the control group. Some spore-forming Clostridia species, such as *Clostridium leptum* and *Clostridium coccoides,* have been involved in the maintenance of the gut mucosa homeostasis by promoting regulatory T cell expansion, attributable to the accumulation of transforming growth factor–β and induction of Foxp3+ transcription factor [41]. Indeed, studies have shown that some *Bacteroides* species, particularly *Bacteroides fragilis*, can drive the development of IL-10-producing Foxp3+ regulatory T cells in the gut mucosa in germ-free conditions [42]. In our study, we reported an increase in IL-4 and IL-10 serum concentrations in RA patients receiving DMARDs. Some previous studies have shown the influence of these DMARDs in cytokine profile, with significant reduction in serum pro-inflammatory cytokines, such as TNF, IL-12, and IL-17, and increased IL-4 and IL-10 concentrations [43,44,45,46].

There are few studies [19,20,36] regarding the influence of specific DMARDs on gut microbiota composition, and some questions should be addressed, including “Do these DMARDs directly influence the gut microbiota composition and their generated metabolities?”, “How do the gut microbes interact with immune cells in the gut mucosa in response to these DMARDs?”, “Is there a specific treatment duration to induce changes in the gut microbiota?”, and finally “Can we offer some specific probiotics that improve the clinical response to DMARDs?”.

## 5. Conclusions

We concluded that gut bacteria are different between RA patients receiving DMARDs and healthy controls. Moreover, DMARDs might be associated with the increased anti-inflammatory cytokines found in RA patients. We also suggest that the gut microbes could be involved in the clinical response to DMARDs. However, further studies are necessary to determine the real role of the gut microbes and their metabolities in clinical response to different DMARDs in RA patients.

## Figures and Tables

**Table 1:** Demographic and clinical characteristics from rheumatoid arthritis patients receiving disease modifying antirheumatic drugs (DMARDs).

Patients	Sex/Age	Ethnicity	DAS28-CRP3	RF (UI/mL)	ESR (mm/h)	CRP (mg/dL)	Disease Duration (years)	Current Treatment
**RA01**	F/64	Caucasian	3.53	ND	46	0	12	PRED, NAP/ESO, SSZ
**RA02**	F/66	Caucasian	4.26	ND	10	1.3	20	MTX
**RA03**	F/37	Caucasian	3.67	8.70	30	1.6	4	NAP/ESO, PRED
**RA04**	F/49	Caucasian	3.03	ND	5	0.73	5	PRED, MTX, LEF
**RA05**	F/53	Hispanic	4.24	9.20	24	1.0	15	DFZ
**RA06**	F/66	Caucasian	4.12	64.0	68	0.6	8	PRED, MTX, ADA
**RA07**	F/55	Hispanic	4.50	41.0	6	0.9	25	MTX, ADA
**RA08**	F/50	Hispanic	3.87	22.7	69	2.2	25	MTX, PRED
**RA09**	F/71	Caucasian	4.65	15.8	9	0.4	15	MTX, PRED
**RA10**	F/59	Caucasian	5.21	932.5	51	2.0	7	ABA, MTX
**RA11**	F/63	Caucasian	4.96	1102.5	99	3.5	10	PRED
**RA12**	F/51	Caucasian	2.65	100.0	63	0	3	MTX
**RA13**	F/64	Afro-descendent	3.71	79.9	68	4.1	12	Meloxicam
**RA14**	F/36	Caucasian	4.34	365.0	31	1.2	14	MTX, PRED, ADA
**RA15**	F/61	Caucasian	2.65	353.2	50	0	12	MTX
**RA16**	F/57	Caucasian	4.89	27.0	72	7.0	2	MTX
**RA17**	F/46	Hispanic	3.58	16.8	34	0.5	12	ADA, LEF
**RA18**	F/62	Hispanic	3.71	55.0	7	0	10	PRED, NAP/ESO, HCQ
**RA19**	F/61	Caucasian	4.84	ND	35	4.0	4	ABA, LEF
**RA20**	F/64	Caucasian	3.95	120.0	48	0	15	PRED

DMARDs: disease modifying antirheumatic drugs; RA: rheumatoid arthritis; F: female; DAS28-CRP3: disease activity score; RF: rheumatoid factor; ND: not determined; ESR: erythrocyte sedimentation rate; mm/h: millimeters per hour; y: years; CRP: C-reactive protein; PRED: prednisone; NAP/ESO: naproxen/esomeprazole; SSZ: sulfasalazine; MTX: methotrexate; LEF: leflunomide; DFZ: deflazacort; ADA: adalimumab; ABA: abatacept; Meloxicam: cyclooxygenase-2 non-steroidal anti-inflammatory drug; HCQ: hydroxychloroquine.

**Table 2:** Description of the main dietary habits of the rheumatoid arthritis patients and healthy controls.

Consumption Frequency	Number of Individuals (N)	RA Patients (%)	Number of Individuals (N)	Healthy Controls (%)	Chi-Squared *p*-Value	Adjusted *p*-Value
**Vegetables**						
Never	-	-	-	-	*p* = 0.676	*p* = 1.000
* Rarely	5	25	6	20		
# Frequently	15	75	24	80		
**Fresh fruits**						
Never	-	-	-	-	*p* = 0.273	*p* = 0.910
* Rarely	5	25	12	40		
# Frequently	15	75	18	60		
**Carbohydrates**						
Never	1	5	1	3.3	*p* = 0.953	*p* = 1.000
* Rarely	5	25	8	26.7		
# Frequently	14	70	21	70		
**Animal-derived proteins**						
Never	-	-	-	-	*p* = 1.000	*p* = 1.000
* Rarely	8	40	12	40		
# Frequently	12	60	18	60		
**Trans fats**						
Never	3	15	6	20	*p* = 0.859	*p* = 1.000
* Rarely	12	60	18	60		
# Frequently	5	25	6	20		
**Milk and derivatives**						
Never	1	5	1	3.3	*p* = 0.957	*p* = 1.000
* Rarely	6	30	9	30		
# Frequently	13	65	20	66.7		
**Hot drinks (coffee/tea)**						
Never	1	5	1	3.3	*p* = 0.102	*p* = 0.51
* Rarely	-	-	6	20		
# Frequently	19	95	23	76.7		
**Canned food**						
Never	7	35	8	26.7	*p* = 0.812	*p* = 1.000
* Rarely	11	55	19	63.3		
# Frequently	2	10	3	10		
**Condiments (ketchup/mayo)**						
Never	8	40	12	40	*p* = 0.46	*p* = 1.000
* Rarely	11	55	18	60		
# Frequently	1	5	-	-		
**Spicy food**						
Never	7	35	15	50	*p* = 0.005	*p* = 0.05
* Rarely	3	15	12	40		
# Frequently	10	50	3	10		

* Less than once a month/1–3 times a month/1–2 times a week; # Most days, but not every day/Every day.

## Abbreviations

RA	Rheumatoid arthritis
PCR	Polymerase chain reaction
IL-4	Interleukin-4
IL-10	Interleukin-10
DMARDs	Disease modifying antirheumatic drugs
RF	Rheumatoid factor
CRP	C-reactive protein
CIA	Collagen-induced arthritis
IL-6	Interleukin-6
IFN-γ	Interferon-gama
IL-17	Interleukin-17
Th17	T helper 17 CD4+ lymphocyte
NSAIDs	Non-steroidal anti-inflammatories
*Bac*	*Bacteroides* species
*Bif*	*Bifidobacterium* species
*Ccoc*	*Clostridium coccoides* species
*CIEub*	*Clostridium coccoides-Eubacteria rectale* subgroup
*Clept*	*Clostridium leptum* species
*Lac*	*Lactobacillus*
*Prev*	*Prevotella*
*Ros*	*Roseburia*
DAS28	Disease activity score
TNF	Tumor necrosis factor alpha
CTLA-4	Cytotoxic T-lymphocyte-associated antigen 4
